# Sclerotherapy needle injections can expand the subserosal and muscularis propria layers and cause a stable mucosal lift in ESD/EMR patients

**DOI:** 10.1007/s00464-018-6521-5

**Published:** 2018-10-22

**Authors:** Jaspreet Sandhu, Carl Winkler, Xiaohong Yan, Abdelsalam Sharabi, Zachary Grimes, H. M. C. Shantha Kumara, Vesna Cekic, Richard Whelan

**Affiliations:** 1 Department of Surgery, Mount Sinai West Hospital, New York, NY USA; 2 Department of Surgery, Brookdale University Hospital & Medical Center, One Brookdale Plaza, Brooklyn, NY 11212 USA

**Keywords:** Bowel wall injections, Mucosal lift, Submucosal lift, Sclerotherapy catheter, ESD, EMR

## Abstract

**Background:**

A mucosal lift is needed for ESD and EMR. Most lifts are made via sclerotherapy needle injection. The firm push needed to penetrate the mucosa often leaves the needle tip in the deep wall. The needle is next withdrawn and fluid injected until a sharp lift (due to submucosal expansion) begins to form; the needle is then held steady and the injection finished. The initial injection may result in a subtle deep lift that resolves quickly. It was the authors’ belief that only submucosal expansion could lead to a stable mucosal lift. A colonic ESD case in which a polyp was inadvertently resected via needle knife in an expanded subserosal plane led to a questioning of this position. This study’s purpose was to determine if stable deep wall mucosal lifts can be generated via bowel wall injection.

**Methods:**

Transmucosal and intramural injections into bovine large bowel were carried out. Stable lifts and lift cross sections were made and examined grossly and histologically to determine the location of the lift fluid. Clinical ESD videos were also reviewed.

**Results:**

Over 200 intact and cross-sectioned lifts were assessed. Gross inspection revealed two types of lifts (superficial and deep), whereas cross sections and histologic analyses revealed examples of stable expansion of the submucosal, muscularis propria, and subserosal layers post injection. Clinical “deep” lifts were also found. Superficial lifts are more focal and taller, whereas deep wall lifts are broader and less prominent.

**Conclusion:**

Stable deep wall mucosal lifts occur and are likely due to the deep starting point of the needle post insertion. If ESD/EMR are attempted with a deep lift, the chances of failure or perforation are high. Lifts must be carefully scrutinized before starting ESD/EMR. Other means of lift establishment should be evaluated and considered.

**Electronic supplementary material:**

The online version of this article (10.1007/s00464-018-6521-5) contains supplementary material, which is available to authorized users.

The creation of a submucosal lift by injecting saline or other solution into the submucosa to expand that space and elevate the mucosal lesion into the bowel lumen is critically important for both EMR and ESD. The resulting submucosal cushion increases the distance between the muscular propria and the mucosal polyp which decreases the risks of perforation when a hot snare or needle knife is used.

In ESD, a mucosal lesion is removed by detaching it from the bowel wall in the submucosal layer after incising the mucosa [1, 2]. After the vertically aligned submucosal attachments have been exposed, with an adequate mucosal lift in place, the scope tip with needle knife extended is moved parallel to the bowel surface and the submucosal fibers are cut. At times, the scope, with a hollow plastic cap attached that extends ½ cm beyond the tip, is fully inserted into the submucosal space placing the attachments on stretch which are then cut (cap dissection) (Fig. 1). In EMR, a snare is used to remove large sessile polyps in a piecemeal fashion [3, 4]. The snare encircles and detaches a portion of the polyp by burning through its attachments to the bowel wall with electrocautery. If the mucosal polyp is well lifted then the snare will only be in contact with submucosal fibers, thus, the deeper muscularis propria is not burned. With a poor lift the muscle layer is at greater risk. Thus, both ESD and EMR can be safely performed only if a submucosal lift is present and the submucosal layer has been expanded.

**Fig. 1:**
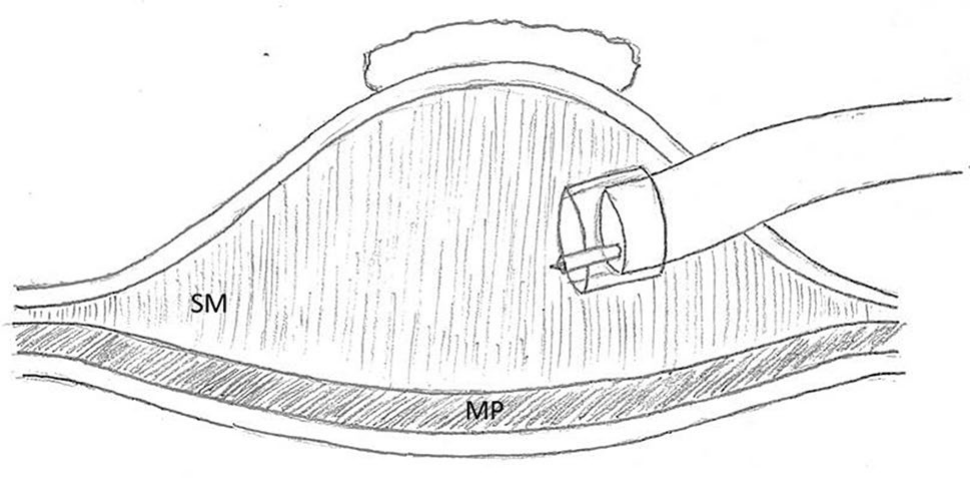
Cap dissection being performed in the submucosal layer. (*SM* submucosa, *MP* muscularis propria)

Currently, submucosal lifts are most commonly established via a sclerotherapy catheter (with retractable needle) passed through the endoscope’s instrument channel and then inserted into the bowel wall. Usually, the needle needs to be pushed with force to penetrate the mucosal surface; a gentle needle push does not puncture the mucosa but simply pushes it aside. The force required to breach the bowel wall most often puts the needle tip beyond the wall altogether or in the subserosal or intramuscular layers. The needle is then slowly withdrawn from the wall as the solution is injected slowly. When the needle is in the submucosal space, the mucosal surface quickly and sharply rises after which the speed and force of injection is increased and the lift established. Not uncommonly, during the initial needle withdrawal, a subtle and broad elevation of the bowel wall is noted in response to the injection. Because it is usually clear that this elevation is not a true submucosal lift, the needle is further withdrawn until the sharp, focal, and more pronounced lift is observed; at this point, a more forceful injection is made. Characteristically, the initial “deep wall lift” dissipates almost immediately after the injection is stopped.

The authors, who have been learning about and utilizing EMR and ESD methods for 8 years, have assumed that if a stable mucosal lift was achieved that the lift solution was in the submucosal space. A case this past year, however, led us to challenge this assumption. The case in question was an ESD of a right colon polyp. After establishing a stable lift, the mucosa was scored, the edge undermined and cap dissection begun in what was thought to be the submucosal plane (Fig. 1). After completing the polypectomy, it was noted that the small bowel could be seen through the intact base of the polypectomy wound (Fig. 2). It turned out that the muscularis propria layer had been fully divided and that the polyp had been detached in the subserosal plane (the layer between the muscularis propria and the serosa) (Fig. 3). A laparoscopy was then carried out (as called for in our current IRB approved protocol to check for a perforation or bowel wall injury post colonic ESD) and the bowel wall imbricated with sutures. Luckily, there was no full thickness perforation. The patient did well postoperatively.

**Fig. 2:**
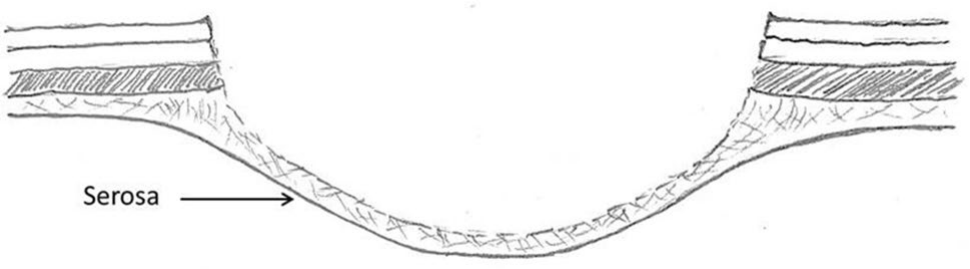
Inadvertent dissection in the subserosal plane leaves behind a thin subserosal layer along with the serosa

**Fig. 3:**
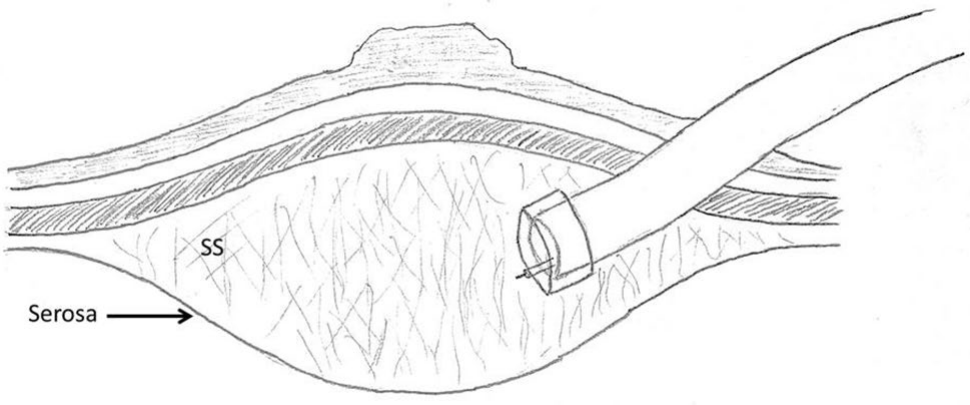
Dissection being performed in the subserosal plane that has been inadvertently expanded by injecting lifting solution. (*SS* subserosal plane)

After this case, we began looking into the concept of a stable deep bowel wall lift. A literature search revealed a single case report regarding a rectosigmoid polyp that was also inadvertently removed in the expanded subserosal plane [5]. The search also revealed two reports concerning gastric cases wherein the subserosal space was intentionally injected in order to (1) perform a full excision of the muscularis propria layer and (2) decrease the perforation risk during gastric EMR [6, 7]. The lift literature otherwise consists of studies comparing different lift solutions; all assess either the duration and/or height of the lifts. We found none that actually confirmed that the lifts were submucosal in location [8, 9]. The hypothesis of the present study was that direct injections into the deeper layers of the bowel wall (subserosal space and/or muscularis propria) during the initial needle withdrawal process may result in an expansion of one or both of these deep layer(s) and a notable elevation of the mucosal surface that persists post injection (Fig. 4). This report presents the results of a series of qualitative bowel wall lift assessments and observations as well as histologic analyses that were carried out in an effort to test this hypothesis.

**Fig. 4:**
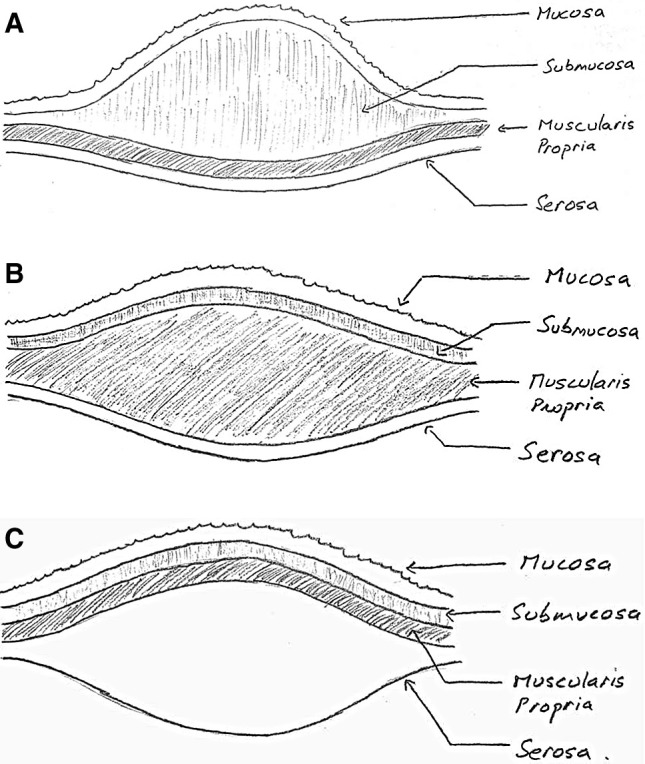
**A** Expanded submucosal layer, **B** Expanded muscularis propria layer, **C** Expanded subserosal layer

## Materials and methods

### Materials

1. Bovine colons used in this study: # IACUC-2016-0007 Icahn School of Medicine at Mount Sinai
2. Human ESD video clips used as supplementary material in the manuscript: IRB HS#: 16-01083 Icahn School of Medicine at Mount Sinai

### Methods

The bowel wall injections for the current experimental study were carried out on ex-vivo bovine large bowel specimens. During the past 6 months the authors have been utilizing bovine large bowel for ESD training, thus, colon specimens were available. After injection, stable intact mucosal lifts were assessed via (1) visual assessment, (2) cross section evaluation after full thickness incision, and (3) histologic analysis. In addition, after a full thickness incision, direct injections were made into the different layers of the cross section in an attempt to expand each space. A brief description of these four methods of assessment follows.

1. Visual assessment: the goal was to intentionally make stable deep and superficial lifts and to characterize each. In some cases an attempt was made to make a more superficial lift on top of an already established lift, a so-called “double lift.” The breadth and height of each lift as well as the ability to see the lift solution dye beneath the mucosa were qualitatively noted. The appearance of the serosal side of the mucosal lift was also noted. Lifts were made with a sclerotherapy needle via colonoscope in intact bovine colon and via mucosal injections made with syringe and needle into flat, full thickness pieces of colon wall (after opening the colon).
2. Cross section evaluation: after making a single or double mucosal lift on a flat piece of colon wall, a scalpel was used to cut directly through the center of the lifted area. The location of the lift solution (submucosal, intramuscular, or subserosal) was noted.
3. Direct injections into the bowel cut edge: after making a 1–2 inch full thickness incision on a flat piece of colon wall, the subserosal, muscularis propria, and submucosal layers were, in sequence, directly injected in an effort to expand each layer.
4. Histologic analyses: a limited number of full thickness square pieces of bowel wall that contained stable mucosal lifts (both superficial and deep) were harvested from the bovine colon and then prepared for paraffin embedding. The first step in this process is fixation of the tissue in formaldehyde for up to a day. Next, the specimen is dehydrated in alcohol to allow paraffin to embed. Next, the alcohol is cleared with xylene after which the paraffin is embedded. Finally, thin sections are made and then stained with hematoxylin and eosin. Not surprisingly, the “lift” does not survive this process. Thus, the final tissue slides do not show a greatly expanded sublayer but, instead, reveal areas where one layer of the bowel wall has been “sheared” or mechanically separated from the adjacent layer. It is by identifying where in the bowel wall the “sheared” zones or spaces are that the pathologist can identify which layer expanded in response to the injection.

The data to be presented include (1) our observations (bovine), (2) still photos and videos, and (3) the histology results. A few clinical videos are also included. The bulk of the data is qualitative and observational assessments. No statistical analysis was done.

## Results

**Fig. 5:**
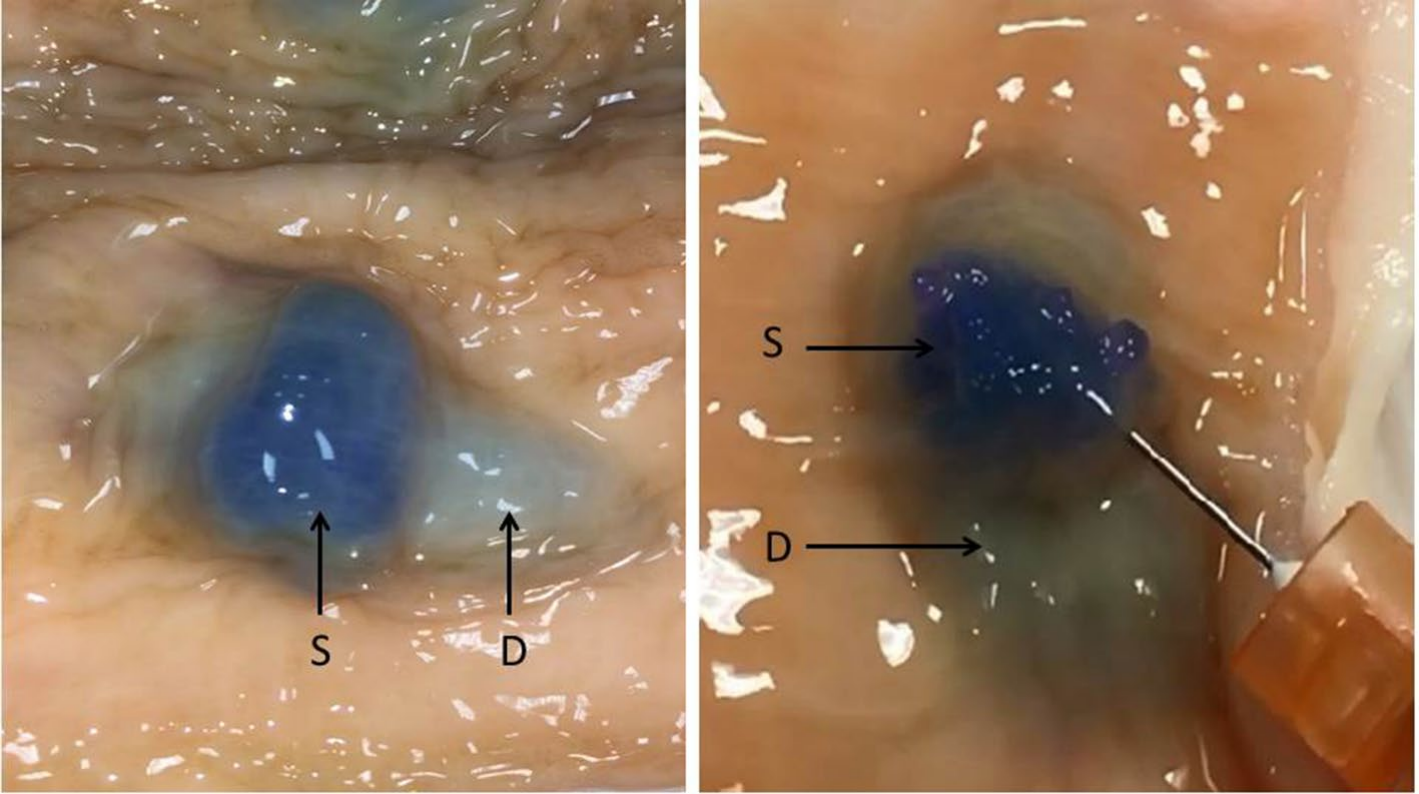
Superficial on deep lift, “Double lift” (*S* superficial lift, *D* deep lift)

**Fig. 6:**
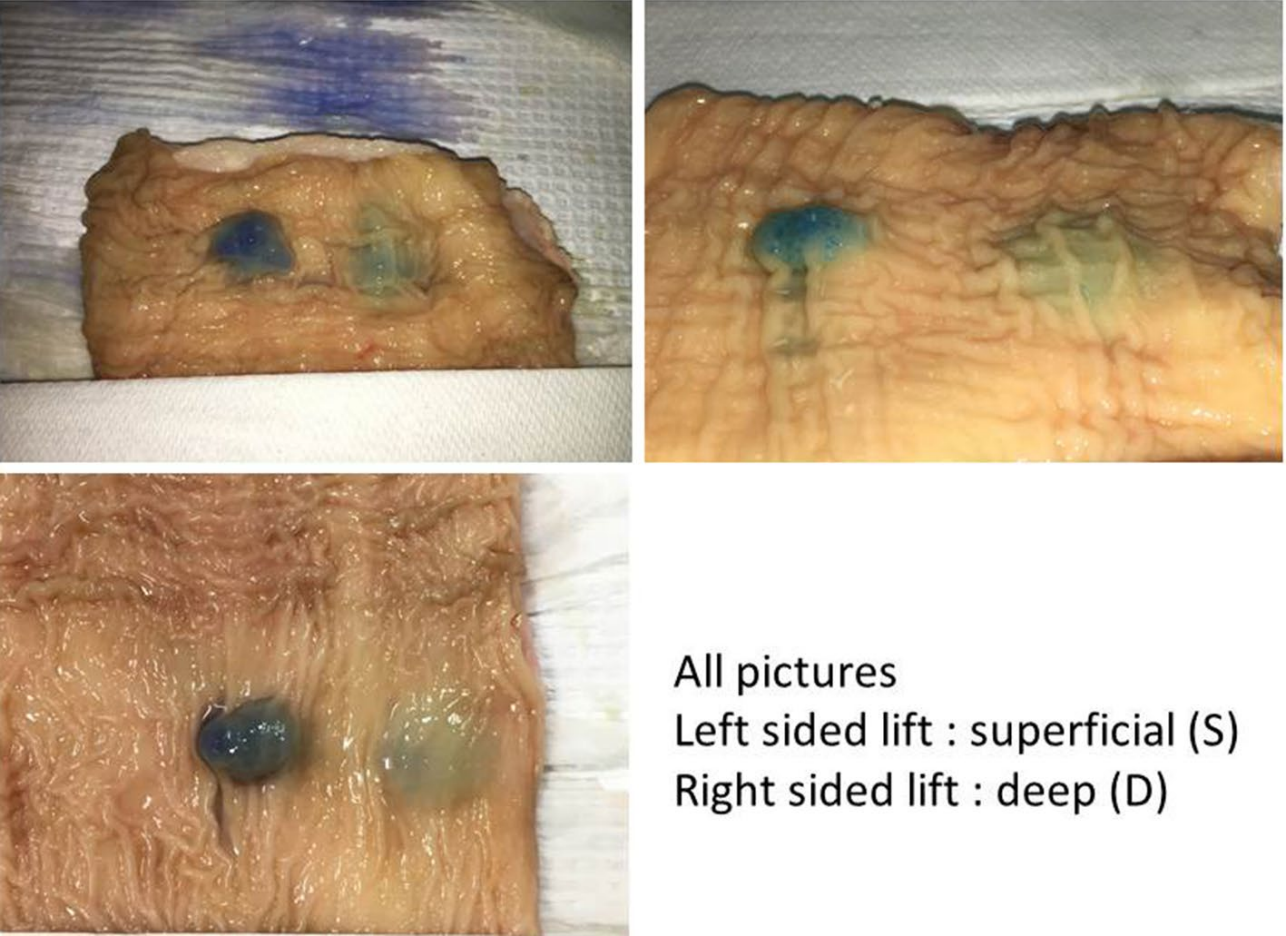
In all pictures, left-sided lifts are superficial (S) and right-sided lifts are deep (D)

**Fig. 7:**
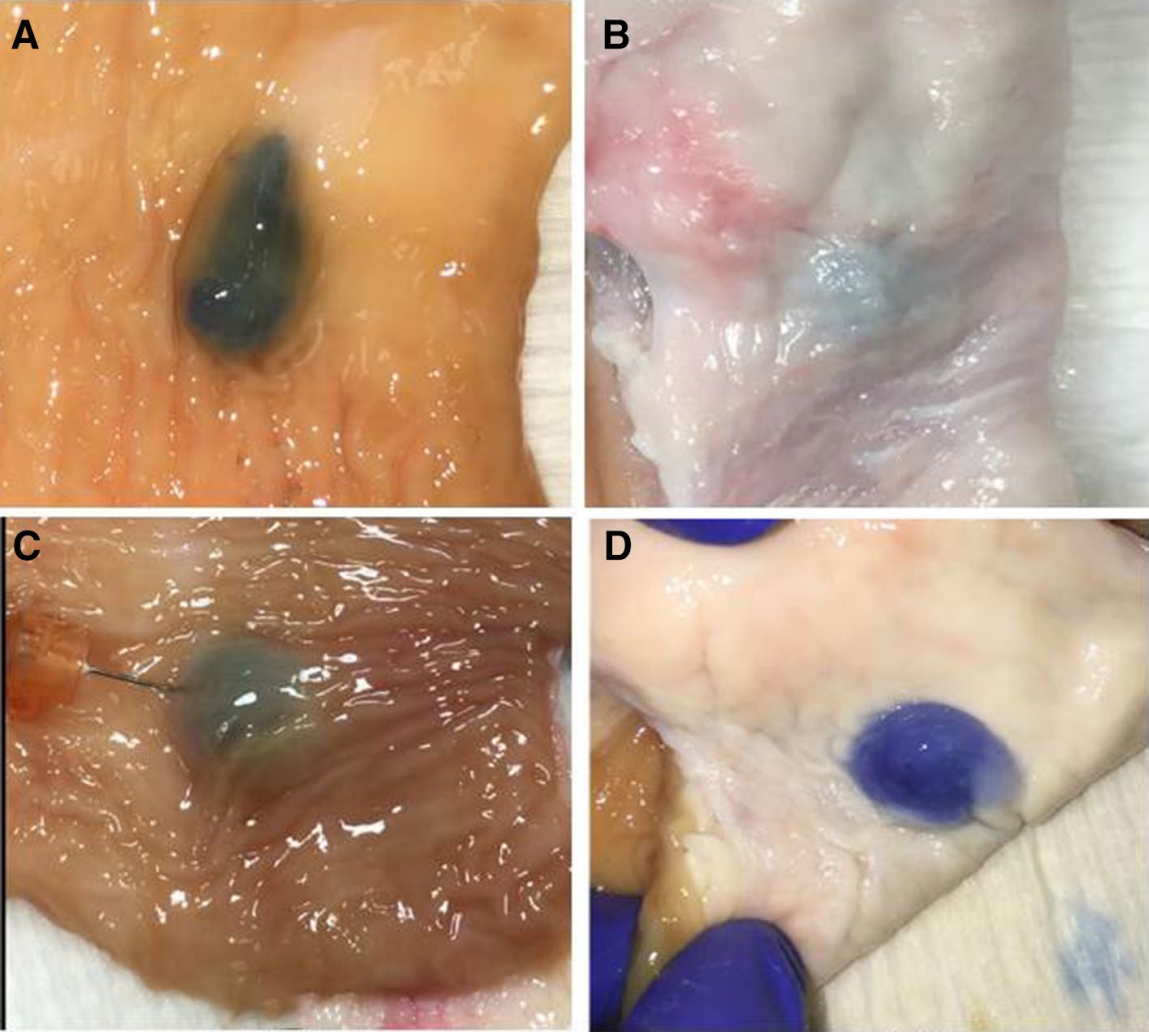
**A** mucosal view of superficial lift, **B** serosal view of superficial lift, **C** mucosal view of deep lift, **D** serosal view of deep lift

Each of the four lift assessment methods provided evidence supporting the “deep lift” hypothesis. Assessment of the intact bowel wall from the mucosal perspective after deep and superficial intramural injections demonstrated there are different types of mucosal lifts with distinct characteristics. Over 200 individual lifts, generated via transmucosal injections in 12 different bovine colorectal specimens, were assessed. The ability to make a distinct superficial lift on top of an already lifted area, the so-called “double” lift, provides the best evidence that deep wall injections can result in stable expansion of the deeper layer(s) of the bowel wall (Fig. 5, Video clips 1, 2, 3). The deep wall lifts were broader and less localized than the superficial lifts that, in contrast, are more distinct and focal. Also, the height of the mucosal elevation is lower in a deep wall lift vs a superficial lift. Another superficial lift characteristic is that the dye color can be readily seen beneath the mucosal surface. In contrast, the color change is either absent or more subtle with deep lifts (Fig. 6). Of note, the serosal appearances of deep and superficial lifts are often different. Uniform dramatic serosal staining is usually noted with deep lifts whereas submucosal lifts may cause no discoloration or subtle light color changes (Fig. 7). Whereas inspection of the bowel after injection suggests that deep lifts can be made, analysis of bowel wall cross sections provides additional evidence.

**Fig. 8:**
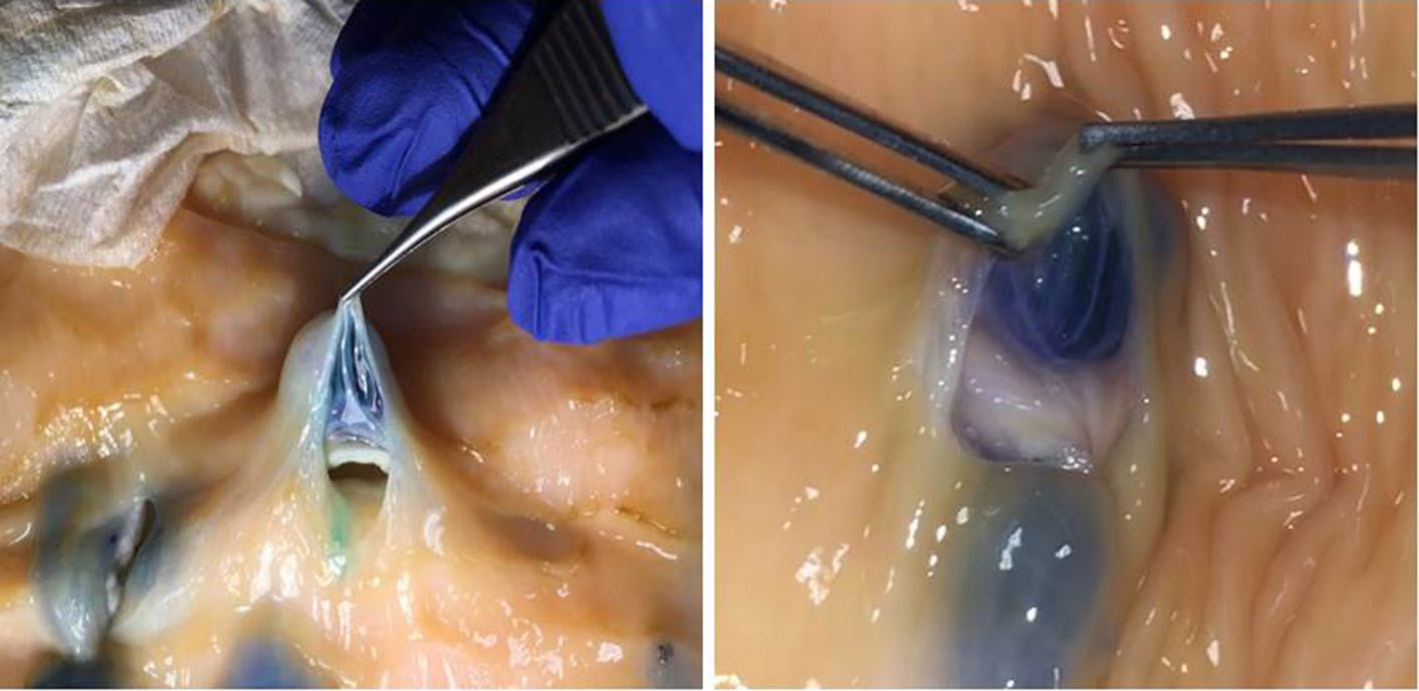
Cross sections showing superficial lifts

**Fig. 9:**
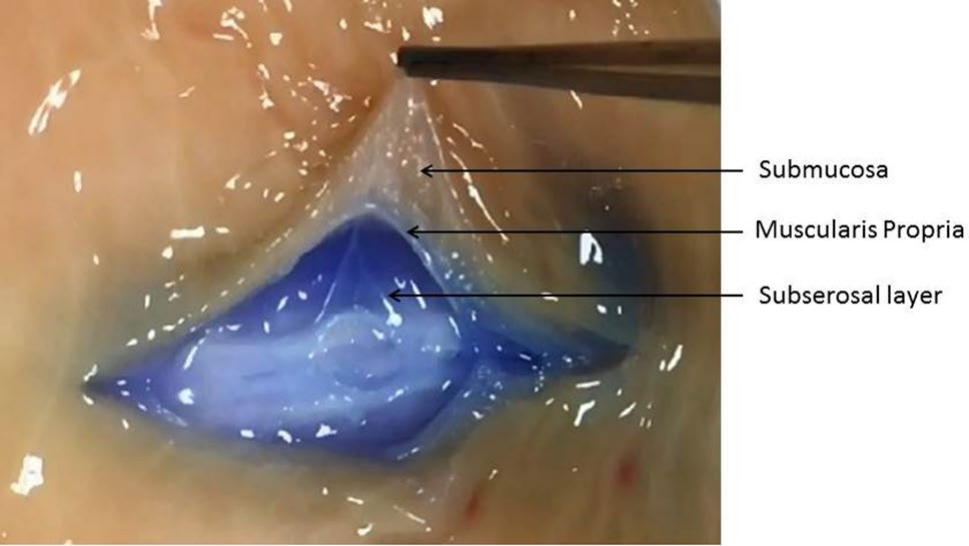
Cross section showing subserosal expansion

**Fig. 10:**
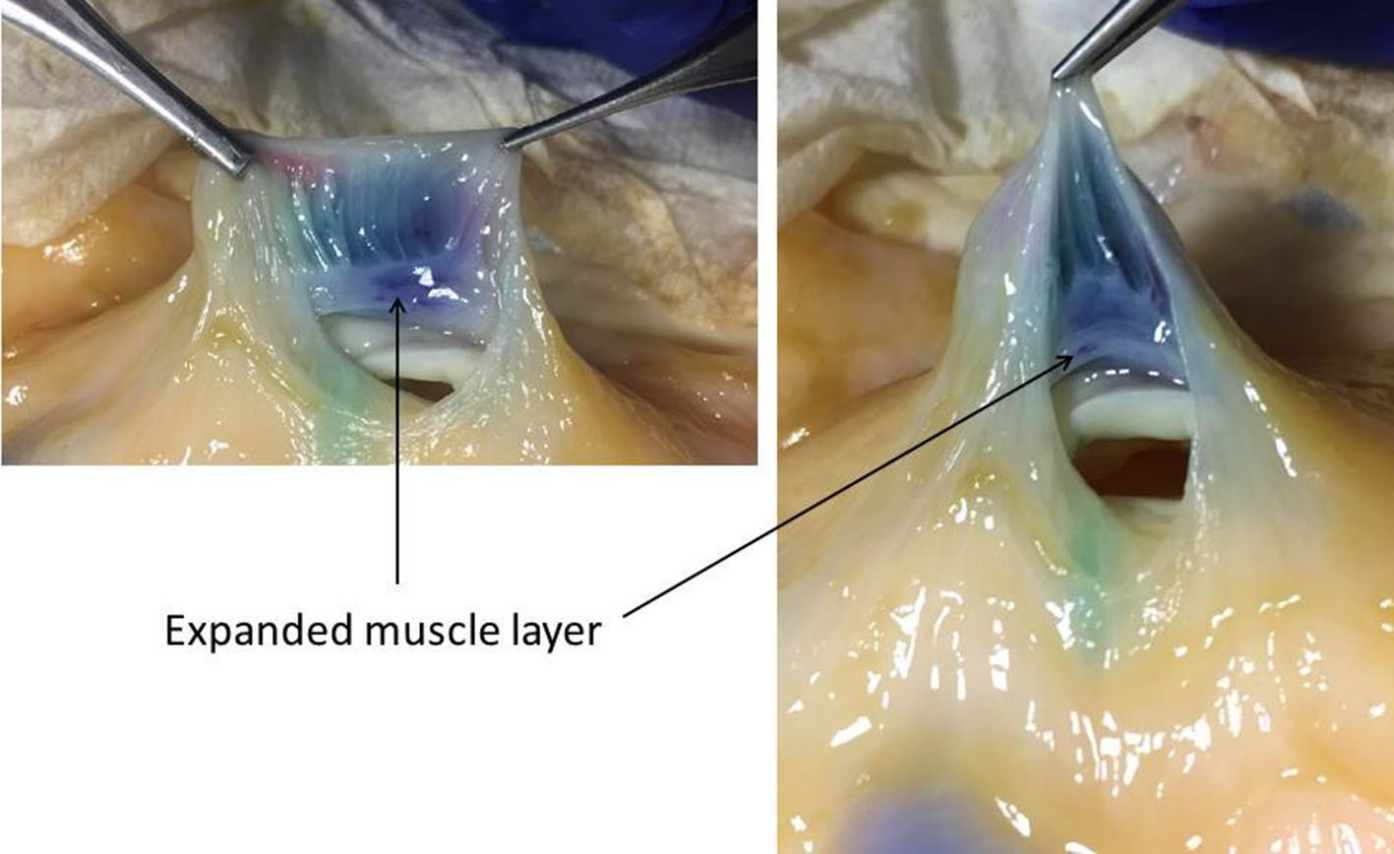
Expansion of muscularis propria layer

Incision through the lifted area soon after injection followed by assessment of the resulting cross section revealed the location(s) of the lift solution (subserosa, muscularis propria, or submucosa). Over 80 lifts were assessed in this way. The dye added to the lift solution greatly facilitated the identification of the bowel wall layers. In cross sections of pristine colon wall, the muscularis propria layer is a thin layer of white fibers. Determining the muscle layers location, either above or below the expanded blue colored layer, is how each lift was characterized. In the majority of lifts examined the solution expanded either the subserosal space (between muscularis propria and serosa) or the submucosal space (between mucosa and muscle layer) (Figs. 8, 9, Video clip 4). In some cases, however, lift solution was found in both spaces. In yet other instances, it was the muscle layer that was clearly expanded (Fig. 10).

**Fig. 11:**
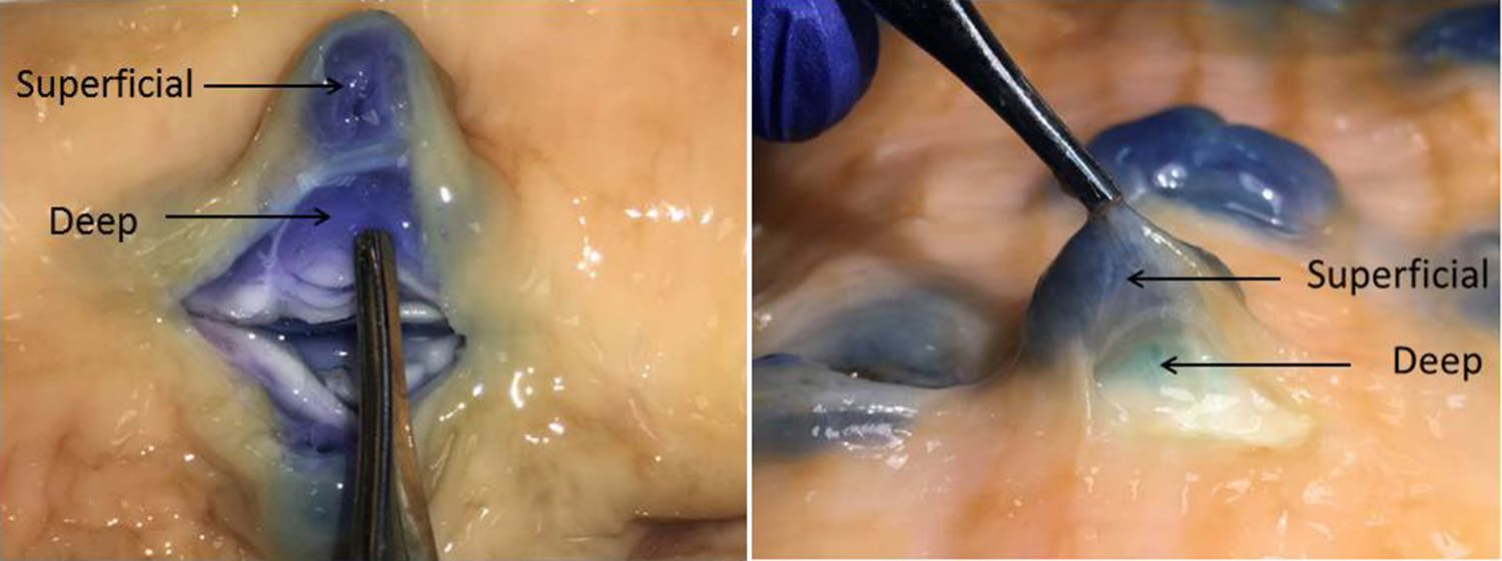
Cross section of double lift (submucosal and subserosal expansion)

**Fig. 12:**
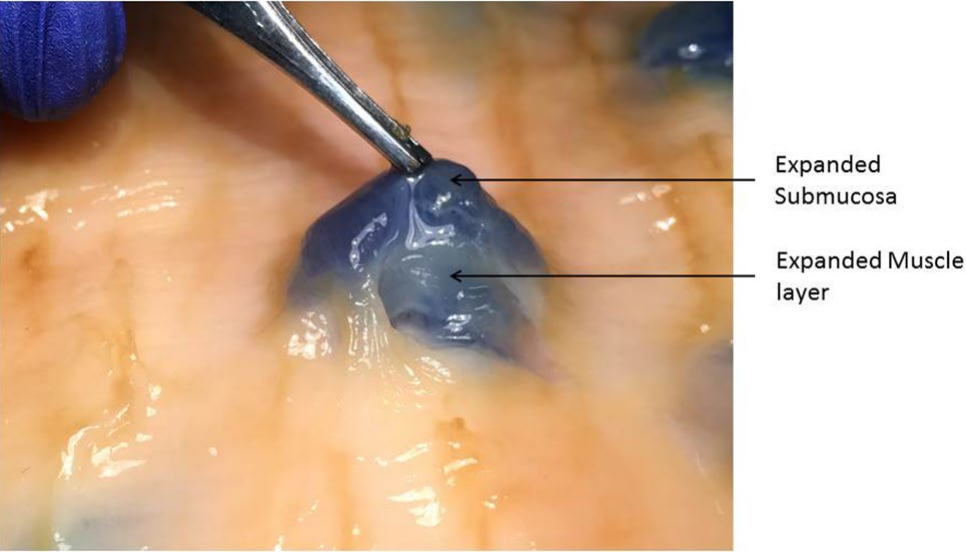
Double lift (submucosa and muscle layer expanded)

The majority of lifts were submucosal in location and were more pronounced and focal. It is important to note that with a subserosal lift the more superficial muscularis propria, submucosal, and the mucosal layers (none expanded) were pushed upwards together into the bowel lumen. Cross sections of “double” lifts again provided the most convincing evidence; in most cases, the lift solution expanded both the layer above and the layer below the muscle layer (Fig. 11, Video clip 5). In some cases, however, the muscle layer appeared to be expanded in addition to the superficial layer (Fig. 12).

**Fig. 13:**
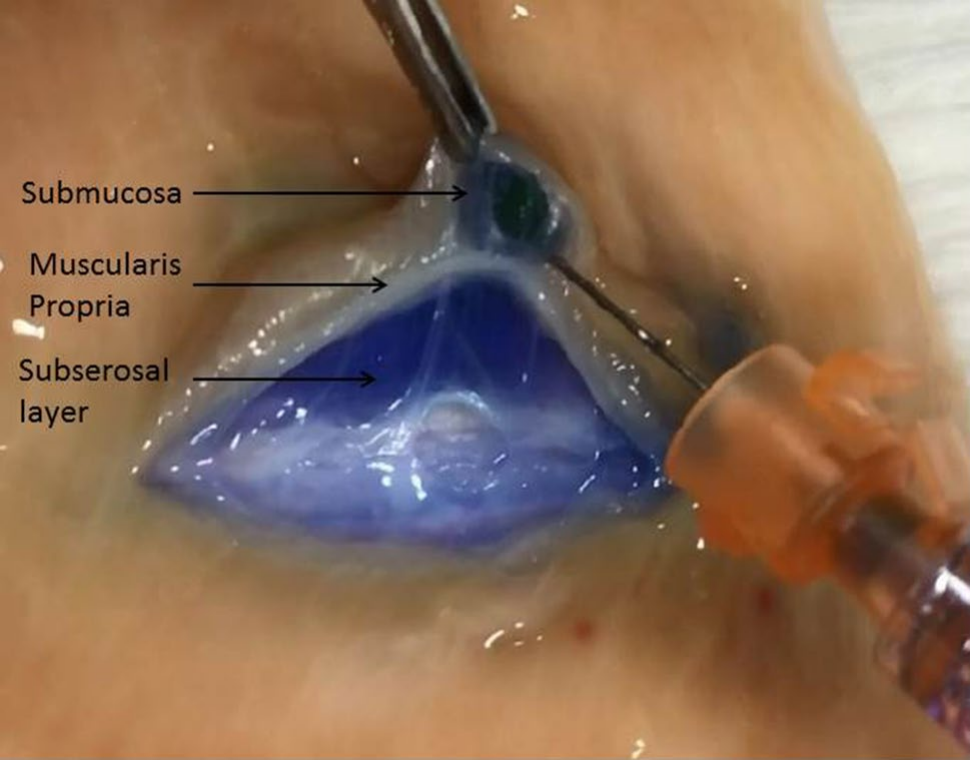
Direct injections into a cut section, “double” lift

Additional deep lift evidence was found when injections were made directly into the individual layers of the cut edge of the bowel wall (injections not via the mucosa). As regards the subserosal and submucosal spaces, direct injection resulted in expansion of the layer in question. Injection of these two layers in succession led to a double lift (Fig. 13, Video clip 6). Also, in a few instances, when the muscularis propria was directly injected it also widened.

Unfortunately, a video recording was not made of the clinical case that led to the authors’ realization that stable deep wall lifts occur. Analysis of other clinical ESD videos, however, revealed an instance wherein slow withdrawal of the sclerotherapy needle after full insertion (in pursuit of the submucosal layer) resulted in an initial subtle but stable lift of the mucosa that was followed by a distinct and more pronounced superficial lift after the needle was further withdrawn (Video clip 7). Several clips from an EMR polypectomy, mid case, also demonstrate deep and superficial lifts (Video clips 8, 9).

### Histologic results

**Fig. 14:**
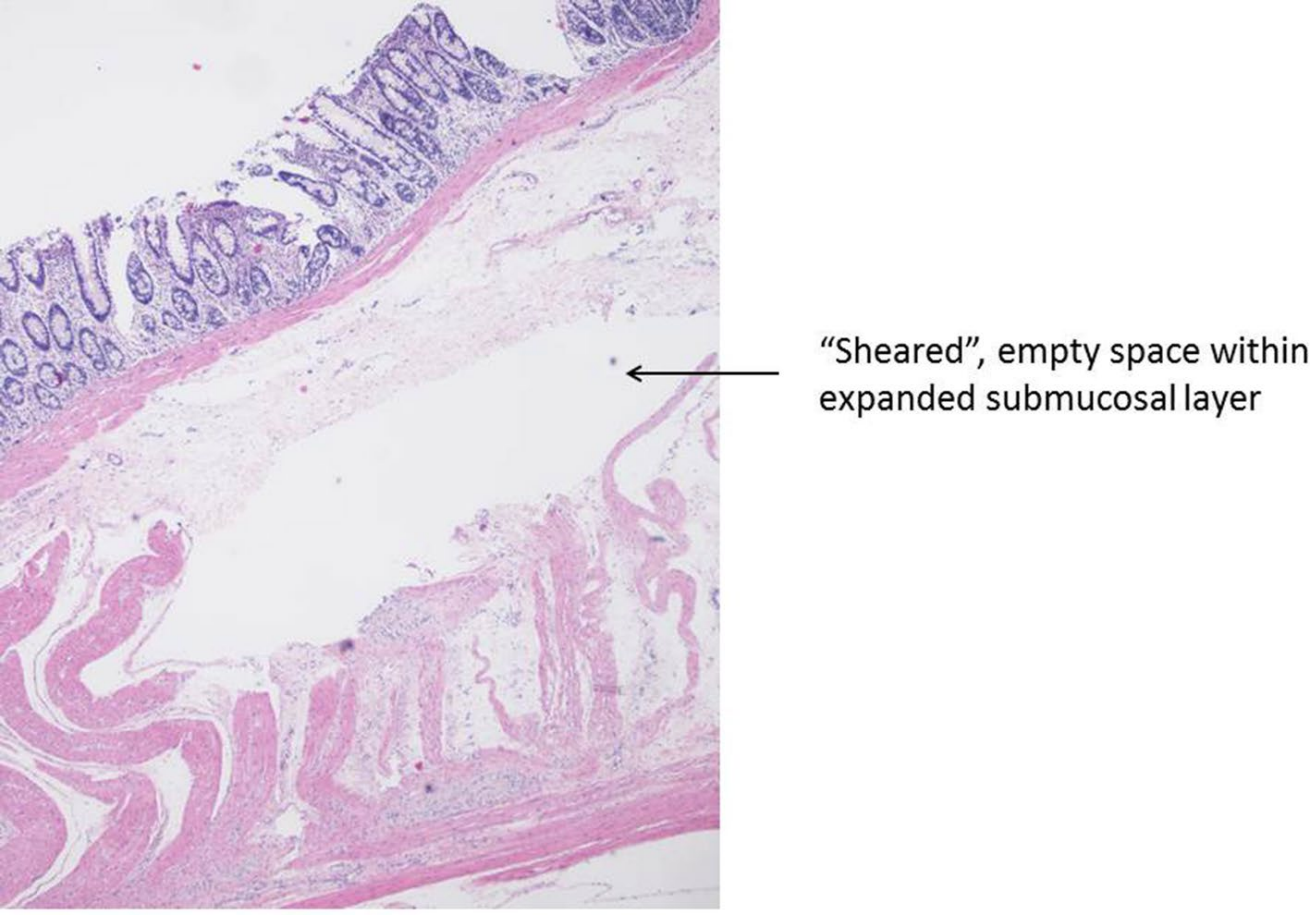
Histologic section showing submucosal separation

**Fig. 15:**
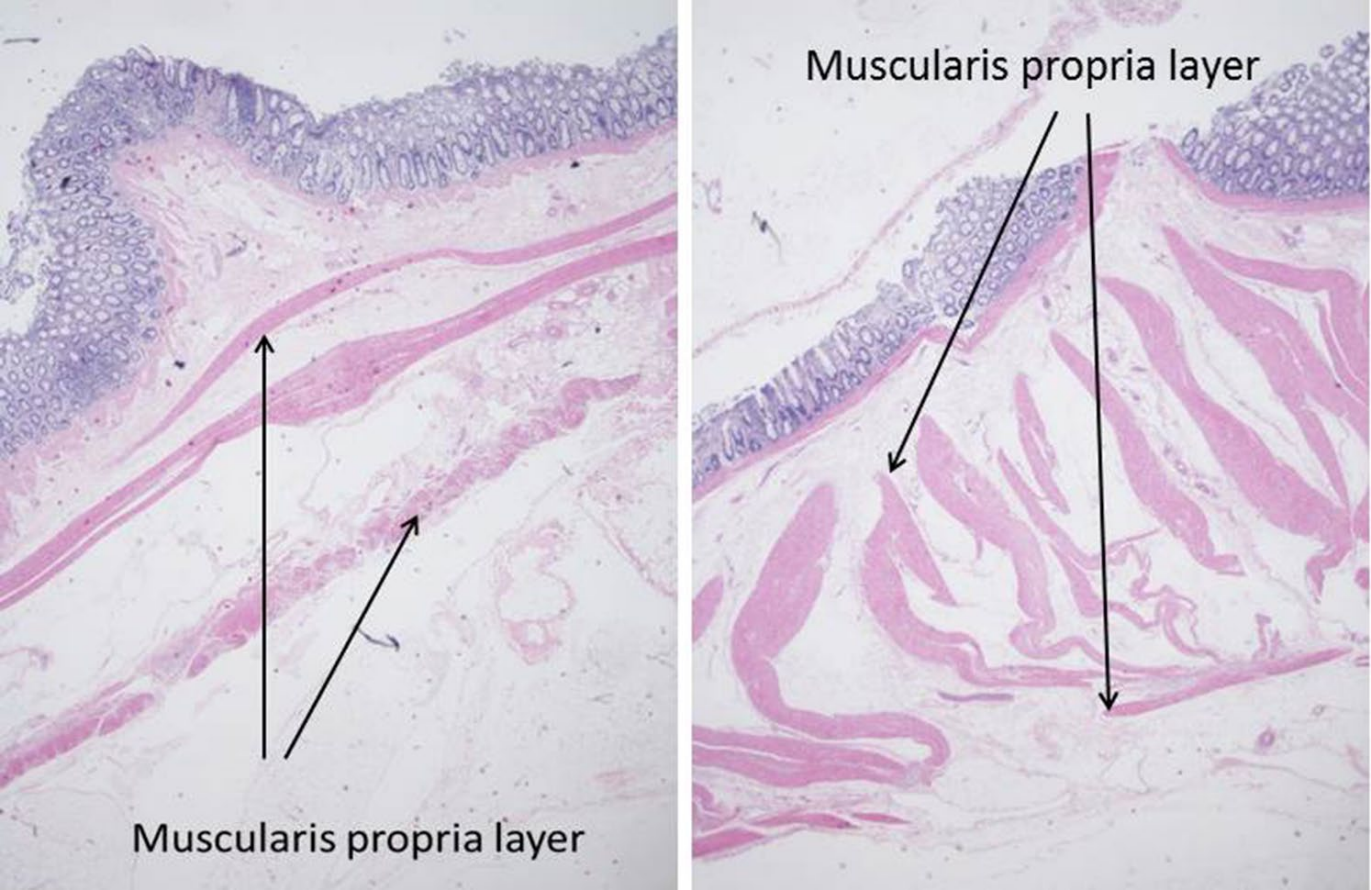
Histologic sections showing the separation of muscularis propria components

**Fig. 16:**
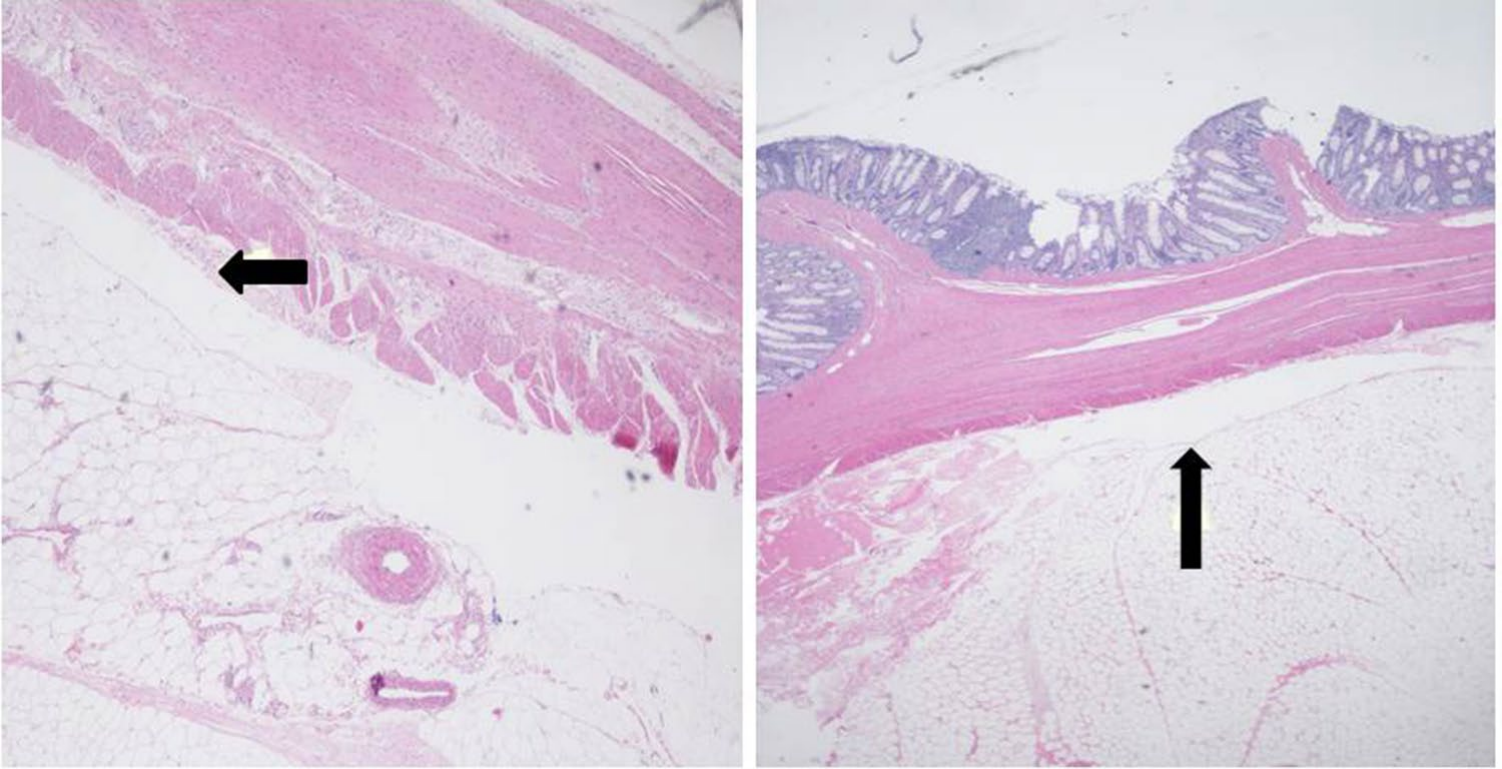
Histologic sections showing subserosal “sheared” empty spaces

**Fig. 17:**
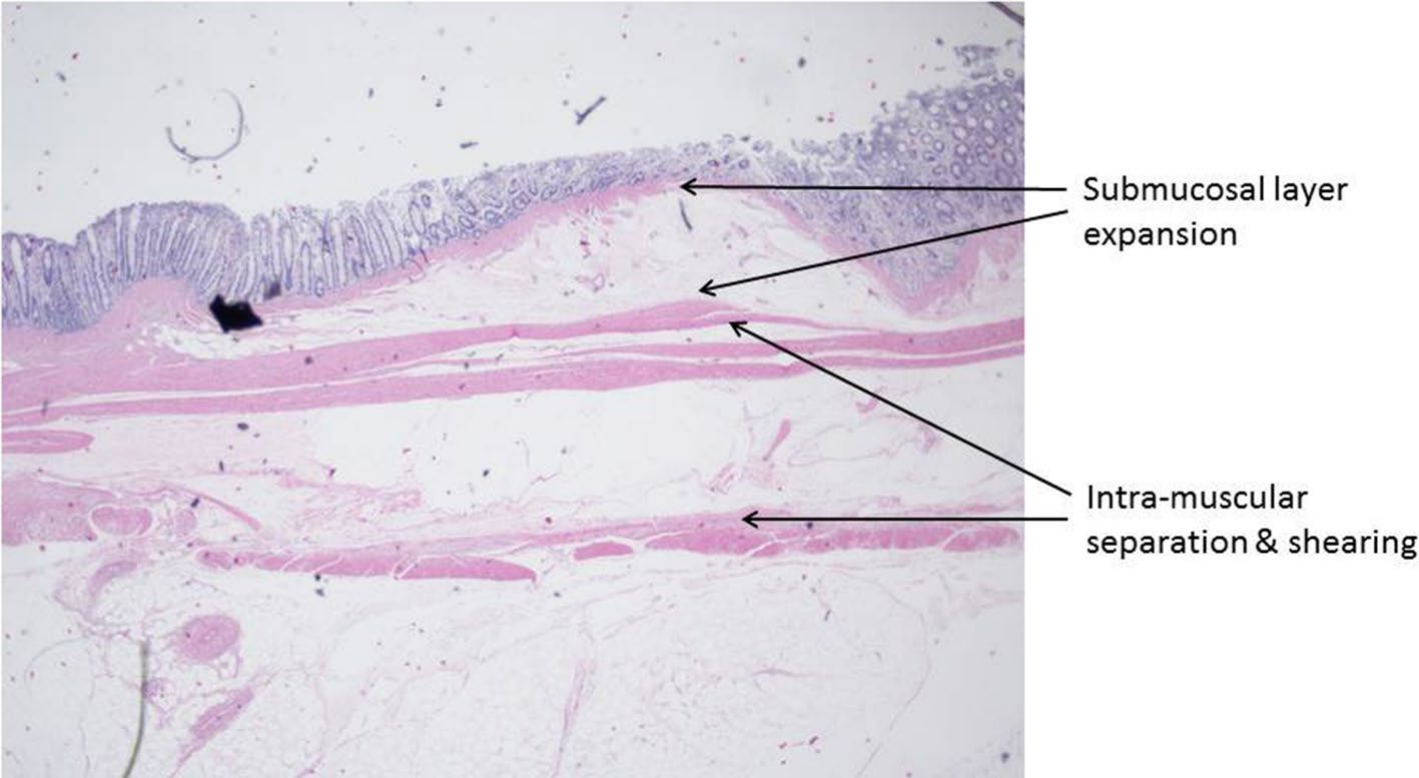
Shearing and expansion of both the submucosal and muscularis propria layers

A limited number of superficial and deep wall stable lifts were histologically assessed by a pathologist. As mentioned in the “Methods” section, injected lift fluid disrupts tissue connections and creates a fluid-filled space which, after tissue preparation, appears as a “sheared” empty zone. The location of the sheared area within the bowel wall reveals the layer that expanded in response to the injection. Amongst the tissue blocks examined, there were examples demonstrating shearing within the submucosal, intramuscular (muscularis propria), and subserosal planes (Figs. 14, 15, 16). In a few tissue sections, there was evidence of shearing in multiple layers (Fig. 17). Given that only full thickness bowel wall segments with stable “lifts” were pathologically processed and assessed, the histologic results confirm that deep wall injections can result in stable expansion of the muscularis propria and subserosal bowel wall layers in addition to the submucosal layer.

## Discussion

To our knowledge, this is the first study to actually determine which layer of the bowel wall expanded in response to transmucosal injections. As mentioned, the relatively small lift literature that exists concerns comparisons of different lift solutions that assessed only the duration and height of lifts [8, 9]. The fact that no one has bothered to document which layer has expanded in these studies suggests that, like the authors, all have assumed that only a submucosal lift could result in a stable mucosal elevation.

From the authors’ perspective, having inadvertently excised a 3-cm sessile polyp in an expanded subserosal plane, it is clear that a stable deep bowel wall lift can be made when using the sclerotherapy needle method of lift establishment. The same conclusion can be drawn from the results of the current study which strongly support the concept that the mucosal layer can be stably lifted in response to deep bowel wall injections.

Close inspection of intact lifts after injection of the bovine bowel wall demonstrates two distinct types of stable mucosal elevations. The first is characterized by a sharp and distinct elevation; in addition, the color of the added dye can usually be seen through the mucosa. A broad and less prominent lift with minimal or no color evident is the second type of mucosal elevation. The attached double lift video clip demonstrates well the two different lifts and the characteristics of each. The cross sections of the established lifts also unmistakably demonstrate that the subserosal, muscularis propria, and submucosal layers can be expanded via transmucosal injections so as to elevate the mucosal surface. The double lift cross sections are especially convincing. Similarly, direct injections into the cut edge of the bowel wall provided yet more evidence that the deep bowel wall layers can expand. It was possible to create submucosal and subserosal blebs similar to those made via transmucosal injections. Less frequently, the muscularis propria layer also appeared to have expanded. Finally, pathologic evaluation of a series of intact stable deep and superficial lifts added yet more evidence that deep wall lifts occur.

Histologic sections of lifted areas strongly suggest that the submucosal, muscularis propria, and subserosal layers are capable of expansion in response to injection. Clearly identifiable spaces within or between these bowel layers, the “sheared” zones thought to represent the areas that expanded in response to injection, were noted in all three of the above layers. Not surprisingly, in some lifts, sheared areas were found in two different layers (example: submucosa and muscularis propria) suggesting that mixed lifts occur. Importantly, the histologic analyses also confirmed our gross visual interpretations of lift cross section anatomy (location of the three layers).

As mentioned, transient deep wall lifts are noted at times when fluid is injected as the sclerotherapy needle is slowly withdrawn from the bowel wall in an effort to find the submucosal plane. The needle tip’s starting point is almost always deep in or beyond the wall because a strong push of the needle is needed to penetrate the mucosal surface. The deep starting point ensures that the initial fluid injected will not be in the submucosal plane. Three variables that likely determine whether a deep wall lift occurs as the needle is withdrawn are (1) the rate of injection, (2) the time the needle tip sits solely in the subserosal or intramuscular planes, and (3) whether or not a serosal puncture was made. If only a small volume is injected, the needle is withdrawn rapidly, or the needle tip resides in several layers simultaneously then expansion of the deep space is unlikely. In rare situations, deep wall expansion does occur and the mucosal surface does rise. However, most often, the lift quickly dissipates post injection. Rapid leakage of lift solution into the peritoneal cavity or mesentery may occur if the serosa has been punctured or if the serosa ruptures in response to bleb formation. Most likely, the very rare stable deep wall lift occurs when the serosa is uninjured, the needle tip is left in the subserosal plane, and a decent volume of fluid is injected. There are several other key variables that influence the chances of successfully lifting the mucosa in either the subserosal or submucosal layers.

In regards to bowel wall injections it should be noted that sclerotherapy needles, either 23 or 25 gage, are sharp and beveled. Because of the bevel, the needle tip has length. To expand the submucosal layer, the entire beveled tip must sit in the submucosal space (for a deep lift in the muscle or subserosal space). Therefore, the sclerotherapy needle angle of entry should be as tangential to the bowel wall as possible. The more acute the insertion angle, the more difficult it will be to place the entire tip in the submucosa. If the needle is partly in the bowel lumen or peritoneal cavity then the fluid will flow into those spaces. The bowel wall thickness is also important; it will be harder to position the needle in a thin wall. Yet another variable is the condition of the bowel wall. It will be hard to establish a lift in irradiated or inflamed bowel because the bowel wall is less compliant and, perhaps, scarred. Also, after a prior polypectomy attempt with a hot snare, scarring between the mucosa and muscularis propria often prevents the submucosal space from expanding. In this situation, a deep lift is more likely because the subserosal plane is intact and the needle tip is more likely to sit in the deep wall because a harder push is needed for insertion.

It is important to note that deep wall lifts are associated with the sclerotherapy catheter/needle method of mucosal lift establishment. It is the need to push the needle fairly hard into the bowel wall in order to penetrate the mucosal surface that results in the needle tip starting out deep to the submucosal layer. A deep starting point ensures that the fluid initially injected will be in the wrong plane. Currently, the alternative means of establishing a mucosal lift is with a needleless high-pressure jet of fluid that is capable of traversing the mucosal surface and expanding the submucosal plane [10]. This high-pressure injection system literally “shoots” fluid into the submucosal space upon contact with the bowel wall but without a needle puncture. A deep wall lift should be far less likely in the absence of a puncture. Up until recently, the only system capable of high-pressure fluid injection required an expensive external pump and a specially designed hollow needle knife that most hospitals did not purchase. Because of these limitations, the use of this technology was limited. Presently, however, there is a high-pressure injection system in the market (and a second on the way) that utilizes a hand-held pump. These new systems do not require an expensive external device and, thus, needleless technology is more likely to be utilized. High-pressure needleless injection technology holds great promise as regards ESD and EMR since it should more reliably establish a submucosal lift. A study of needleless technology is needed to determine if and how often deep wall lifts occur.

It is likely that despite the advent of needleless lift technology that the sclerotherapy needle method will continue to be used in many centers to establish mucosal lifts. Awareness of deep wall lifts leads to close lift scrutinization prior to applying a hot knife or snare. The authors now routinely characterize each lift as deep or superficial and seek a more superficial plane if a deep lift is suspected. Also, after making the initial mucosal incision if a clear superficial submucosal plane is not found, the lift is re-evaluated and additional, more superficial, injections made. Intriguingly, the fact that the subserosal layer and, perhaps, at times, the muscularis propria layer can expand may provide a means of decreasing the risk of perforation during ESD and EMR. In conjunction with a submucosal lift, intentional thickening of the subserosal or muscle layer should decrease the chances of perforation by increasing the distance between needle knife or snare and the serosa. It is now the practice of the authors to make intentional deep wall injections into the exposed muscle surface during the latter stages of ESD, when the submucosal lift is harder to maintain and is not robust.

## Summary

When endoscopic bowel wall injections are carried out with a sclerotherapy catheter/needle prior to ESD or EMR, rarely, stable deep wall lifts develop. This occurs, most often, when the lift solution is inadvertently injected into the subserosal or muscularis propria plane. Deep lifts are less prominent and broader with gently sloping sides versus submucosal lifts which usually are well demarcated and have well-defined edges and fairly steep sides. Also, whereas the dye color in the solution can often be seen beneath a submucosal lift, the dye is usually not seen with a deep lift. It is important to assess each lift critically before starting an ESD or EMR case. If there is a suspicion that the lift might be a deep one then an additional injection should be made after an attempt is made to find a more superficial plane.

## Electronic supplementary material

Below is the link to the electronic supplementary material.

Supplementary material 1 (MOV 8182 KB)

Supplementary material 2 (MOV 7005 KB)

Supplementary material 3 (MOV 15787 KB)

Supplementary material 4 (MP4 14356 KB)

Supplementary material 5 (MOV 7624 KB)

Supplementary material 6 (MOV 6590 KB)

Supplementary material 7 (MOV 10488 KB)

Supplementary material 8 (MOV 22448 KB)

Supplementary material 9 (MOV 12378 KB)
